# Inspector-General Coppinger

**Published:** 1910-04-30

**Authors:** 


The late Inspector-General Coppinger, R.N., M.D., who died recently from a motor-bicycle accident, more than twenty years ago held the post of Fleet-surgeon, conducting instruction to surgeons on entry. His knowledge of the internal economy of ships from a hygienic point of view was great, and he worked both as naturalist and bacteriologist. He took part in the Arctic expedition of 1875-76, and was awarded the Arctic medal. Dr. Coppinger was author of "The Cruise of the 'Alert' in Patagonian and Polynesian Waters."

